# MicroRNA-Target Binding Structures Mimic MicroRNA Duplex Structures in Humans

**DOI:** 10.1371/journal.pone.0088806

**Published:** 2014-02-13

**Authors:** Xi Chen, Lu Shen, Hui-Hsien Chou

**Affiliations:** 1 Department of Genetics, Development and Cell Biology, Iowa State University, Ames, Iowa, United States of America; 2 Department of Biostatistics, Johns Hopkins University, Baltimore, Maryland, United States of America; 3 Department of Computer Science, Iowa State University, Ames, Iowa, United States of America; The University of Tennessee Health Science Center, United States of America

## Abstract

Traditionally, researchers match a microRNA guide strand to mRNA sequences using sequence comparisons to predict its potential target genes. However, many of the predictions can be false positives due to limitations in sequence comparison alone. In this work, we consider the association of two related RNA structures that share a common guide strand: the microRNA duplex and the microRNA-target binding structure. We have analyzed thousands of such structure pairs and found many of them share high structural similarity. Therefore, we conclude that when predicting microRNA target genes, considering just the microRNA guide strand matches to gene sequences may not be sufficient — The microRNA duplex structure formed by the guide strand and its companion passenger strand must also be considered. We have developed software to translate RNA binding structure into encoded representations, and we have also created novel automatic comparison methods utilizing such encoded representations to determine RNA structure similarity. Our software and methods can be utilized in the other RNA secondary structure comparisons as well.

## Introduction

MicroRNAs (miRNAs) are small noncoding RNAs about 21 nucleotides (nt) in length that regulate gene expressions. miRNAs target messenger RNAs (mRNAs) and trigger either their translational repression or degradation [1]–[4]. After pre-miRNA processing, mature miRNA duplexes are loaded into the Argonaute (Ago) proteins within the RNA-Induced Silencing Complex (RISC) [5]–[7]. The assembly activates RISC, which is then directed to target mRNAs [8]–[10]. Before RISC assembly, small RNA duplexes, which include both miRNAs and small interfering RNAs (siRNAs) in some species, can be sorted according to their structure features [11]–[14]. This sorting process is species-specific and determines into which Ago protein will each duplex be loaded. In *Drosophila*, if mismatches happen on the central 9–10th locations of a guide strand counted from the 5′ end, the small RNA duplex will be loaded into its Ago1 protein [11]. Otherwise, it will be loaded into *Drosophila* Ago2 protein [11]. However, it has not been observed that human has a strict small RNA sorting system. Loading of miRNAs into human Ago proteins seems random but predictable — among the 4 human Argo proteins, Ago2 is the most abundant and it interacts with the majority of miRNAs (60%) [15], [16]. A recent study showed that the human miRNA hsa-miR-451 only associates with Ago2 rather than the other Ago proteins [17], which suggested that there may be some human miRNA sorting rules yet to be discovered.

After loading, the unwinding of small RNA duplexes in RISC goes through two different pathways that are either slicer-dependent or slicer-independent [18]–[20]. Slicer-dependent unwinding, which cleaves the passenger strand, requires extensive base-pairing. Human Ago2, the only human Ago protein known to have slicer activity, can cut a perfectly base-paired passenger strand between the 10–11th locations [15]. The nicked passenger strand is then degraded. Most of the human miRNA duplexes have mismatches in this location, i.e., their guide and passenger strands are not perfectly complementary [1], [21]. Therefore, most human miRNAs will instead go through the slicer-independent unwinding [22]. Comparing to the slicer-dependent unwinding, the slicer-independent unwinding is much slower at producing activated RISCs [18].

In the past few years, there were extensive studies on the human miRNA target recognition mechanism. Most of these studies were based on sequence comparisons [23]–[25]. However, considering just miRNA matches to mRNA sequences is not sufficient to correctly predict miRNA targeting sites, resulting in many false-positive candidates [26]–[28]. The miRNA-RISC assembly process has been shown to determine the inhibition efficiency that a miRNA can exert on its target genes [18]. It is not clear how the assembly process affects the inhibition efficiency. It could be that the assembly efficiency determined the amount of activated RISC complexes that can inhibit the target genes, or it could be that the assembly process determined the guide strand structure whose efficiency in binding to target genes is governed by thermodynamics. We hypothesize that the miRNA-RISC assembly process affects the miRNA-target recognition efficiency at the guide strand structural level, i.e., miRNA target prediction precision may be improved by considering sequence matches as well as RNA structure comparisons.

In this work we have tested our hypothesis by studying the correlation of known miRNA duplex structures with known miRNA-target binding structures. All selected miRNA duplexes have experimentally validated guide strand and passenger strand sequences, allowing their duplex structures to be inferred. All selected miRNA-target binding structures have also been experimentally validated. The selected miRNA duplexes were then precisely paired with the validated miRNA-target binding structures — the same miRNA guide strand must be shared between the two structures in any pair. The similarity between the paired structures was then measured by a common RNA structure comparison method, and we have found that 69% of the pairs exhibit high similarity. An alternative statistical measurement was also applied and it revealed that most paired structures share common structural patterns that could not have been generated randomly. Further analysis indicated that this close resemblance of miRNA-target binding and miRNA duplex structures is unrelated to the evolutionary age of the miRNAs. Therefore, our study concludes that the miRNA-target binding structure closely mimics the miRNA duplex structure, and this relationship may need to be considered to improve miRNA target prediction.

## Results

### Selection of paired miRNA duplexes and miRNA-target binding structures

**Figure 1:**
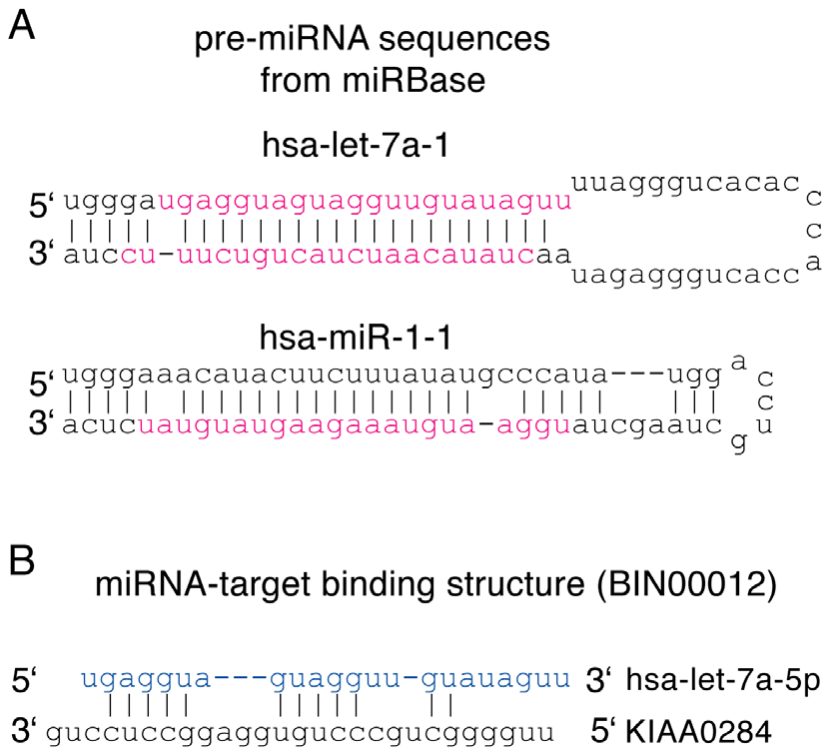
Examples of miRNA structure selections. (A) miRBase includes mature miRNAs with either two known strands like hsa-let-7a-1 (2 red sequences) or only one known strand like hsa-miR-1-1 (1 red sequence). To infer the miRNA duplex structure, we have selected only mature miRNAs with both strand sequences. (B) miRNA-target interaction data were obtained from the paper of Helwak *et al.* In this example, *hsa-let-7a-5p* is the guide strand of hsa-let-7a-1 (colored blue), and its validated binding with one of its targets, KIAA0284, has the structure as shown.

Since our purpose is to compare the structure similarity between miRNA duplexes and miRNA-target binding structures, it is required to collect these structures and pair them together for this study. Human miRNA duplex sequences were selected from release version 18 of the public database miRBase, which is the most comprehensive miRNA database and includes 1527 published human miRNA duplexes (http://www.mirbase.org) [29]. Some mature miRNA duplexes in this database have experimentally verified sequences of both strands but others have only a single verified strand (Figure 1A). To infer the duplex structure of a mature miRNA, we need both strand sequences. As a result, 547 mature miRNA duplexes having both strand sequences were selected from miRBase (Table S1). Human miRNA-target binding structures were selected from the experimental results of Helwak *et al.* [30]. They provided a high confident data set of 18502 miRNA-target binding structures from 413 miRNAs (Figure 1B and Table S2). Because each miRNA normally have multiple target genes, the set of miRNA-target binding structures is significantly larger than the number of participating miRNAs.

**Figure 2:**
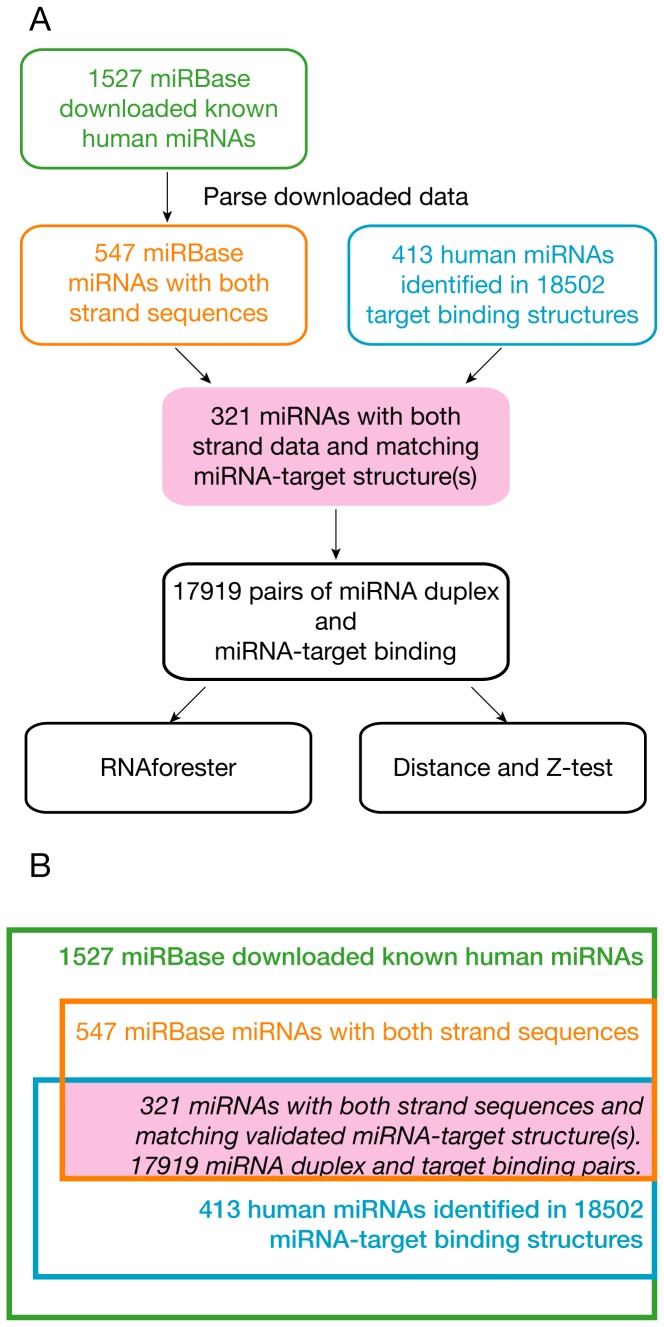
Selection of miRNA duplexes and miRNA-target binding structures for pairwise comparisons. (A) The data selection workflow of miRNA duplexes and miRNA-target binding structures from miRBase and the paper of Helwak *et al.* (B) The selection criteria and the resulted numbers of miRNA duplexes and miRNA-target binding structures. At the end, 321 miRNAs with both strand sequences and validated miRNA-target binding structures were selected and 17919 miRNA duplex and miRNA-target binding pairs were selected for pairwise comparisons.

Intersecting the set of 547 miRNA duplexes having both strand sequences and the set of 413 miRNAs forming the 18502 validated miRNA-target binding structures produced 321 *unique* miRNA duplexes (Figure 2 and Table S3). The *uniqueness* here refers to the duplex structure, not the name of a duplex. For example, the longer pre-miRNA sequences of hsa-let-7a-1 and hsa-let-7a-3 are different, thus their different names, but their shorter mature miRNA duplexes are the same; both have the same guide strand *hsa-let-7a-5p* and the same passenger strand *hsa-let-7a-3p*. To avoid redundancy, only one of the duplicated mature miRNA duplexes was retained in the 321 unique miRNA duplexes. The guide strand within a miRNA duplex was determined according to the miRNA-target binding structure data — the strand that binds to target mRNAs was identified as the guide strand.

After the 321 unique miRNA duplexes were identified, the miRNA-target binding structures associated with them were also selected. Several miRNA duplexes have the same guide strand but different passenger strands (they are considered different among the 321 duplexes). The miRNA-target binding structures associated with the same guide strand were thus paired to all these miRNA duplexes. For example, the guide strand *hsa-let-7a-5p* has 310 different target bindings (Table S2). *hsa-let-7a-5p* is shared by two different miRNA duplexes hsa-let-7a-1 and hsa-let-7a-2. Therefore, 620 structure pairs, the first 310 associated with hsa-let-7a-1 and the other 310 associated with hsa-let-7a-2, were produced. In total, 17919 miRNA duplex and miRNA-target binding structure pairs were produced (Figure 2 and Table S4).

### Global features of miRNA duplexes and miRNA-target binding structures

As mentioned in the Introduction, the way a small RNA duplex will unwind within RISC depends on its mismatch locations. If mismatches happen on the central 8–12th locations of its guide strand, the small RNA duplex will follow the slicer-independent unwinding pathway. Alternatively, if its passenger strand is perfectly complementary to its guide strand, the small RNA duplex will take the slicer-dependent unwinding pathway [18]–[20]. Therefore, mismatch patterns on the guide strand generally have been used in RNA duplex studies [18], [31]–[33]. However, considering only mismatches on the guide strand ignores subtle signals on the passenger strand that may also be important. For example, the pre-miRNA hsa-miR-1-1 on Figure 1A has 2 mismatched adenines on its guide strand shown in red color. When counting only mismatches on its guide strand, both adenines will be counted as 1 mismatch despite their different mismatch patterns on the passenger strand. Therefore, to better compare the global features of miRNA duplexes and miRNA-target binding structures, we have decided to consider the *base-paired* locations on *both* strands.

**Figure 3:**
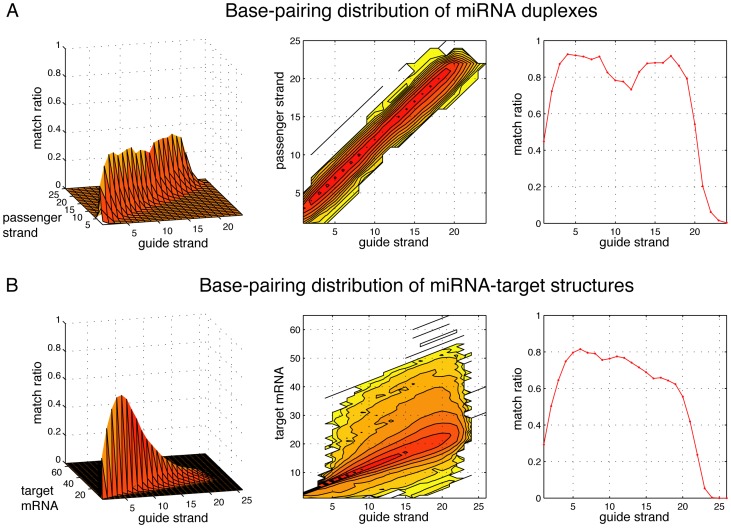
The base-pairing distribution of miRNA duplexes and miRNA-target binding structures. (A) The distribution corresponding to the 321 miRNA duplexes that is drawn in a 3-D surface graph, a 2-D contour map and a 1-D projection onto the guide strand locations. (B) The distribution corresponding to the 18502 miRNA-target binding structures obtained from the Helwak *et al.* paper that are drawn in similar graphs.

Figure 3A illustrates the base-pairing distribution of the 321 miRNA duplexes we have selected. The distribution can be visualized in 3 different ways: a 3-D surface graph, a 2-D contour map and a 1-D projection of the base-pairing counts onto the guide strand axis. Note that the 1-D projection adds up all base-pairing counts along the passenger strand axis that are projected onto the same guide strand location, thus the height of the 1-D projection is higher than the 3-D graph. The similar set of graphs obtained from the 18502 miRNA-target binding structures is provided in Figure 3B. We can see that on miRNA duplexes there is a noticeable dip of base-pairing counts roughly in the central 10–13th locations of the guide strand. The guide strand seed region (the 2–7^th^ nucleotides) has lower base-pairing counts among miRNA-target binding structures than among miRNA duplexes; most base-pairing counts in this region is below 80% for miRNA-target binding structures. This supports the notion that seed region interactions with targets are not canonical for many miRNAs [30]. The other locations on the miRNA-target binding structures also have lower base-pairing counts than the duplexes, but their counts remain high at over 60% throughout most of the structures.

The analysis above supports the validity of our method to count the *base-paired* locations on *both* strands instead of the mismatched locations only on the guide strand, because it reveals many important miRNA features as follows. Among miRNA-target binding structures, the seed region of the guide strand seems to have mixed canonical and non-canonical match patterns, which is consistent with existing knowledge [34], [35]. In addition, miRNA duplexes tend to have lower base-paring counts in its central region, which is also consistent with previous studies [36], [37]. Noticeably, non-seed region base-pairing counts are significantly lower on miRNA-target binding structures than on miRNA duplexes. A possible explanation is that a miRNA must be packaged well in double-stranded format within pre-miRNA before it can be successfully processed and incorporated into RISC. Significant mismatches anywhere along the duplex can disturb the stability of its structure and ruin its function. However, once incorporated into RISC, the miRNA guide strand does not require perfect base-pairing beyond the seed region to target genes. The global features of miRNA duplexes and miRNA-target binding structures seem to suggest that they are not very different in the seed region but are noticeably different in the non-seed region. What we need to find out next is if such distinction holds at the individual structure pair level.

### Pairwise structural comparisons between miRNA duplexes and miRNA-target bindings

**Figure 4:**
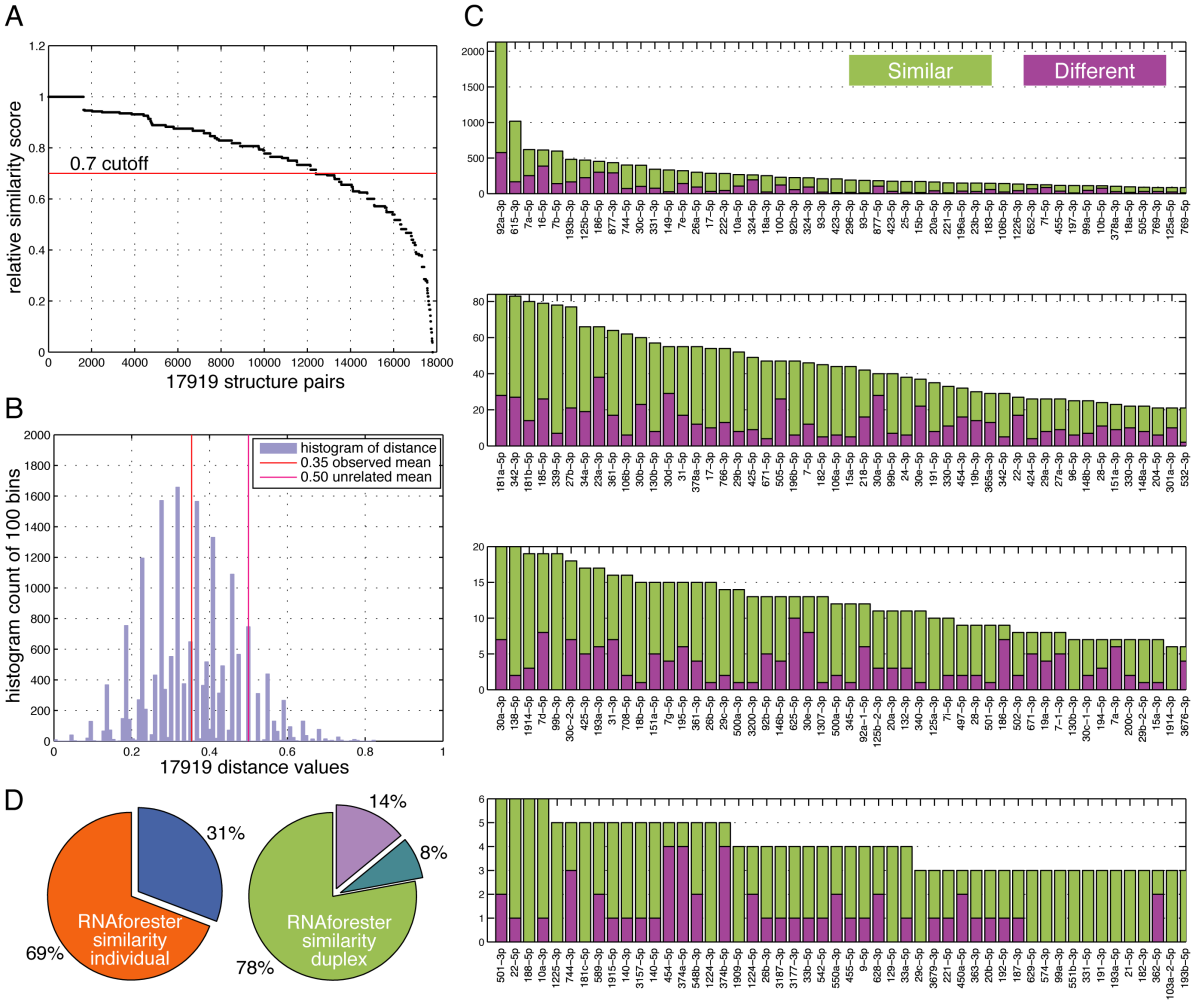
Pairwise comparison of miRNA duplexes and miRNA-target binding structures. (A) The similarity scores of the 17919 structure pairs were calculated by RNAforester and ordered from high to low; scores above 0.7 are considered similar. (B) The histogram of the length-scaled Hamming distance values of the 17919 structure pairs divided into 100 bins (bar chart) with the mean value at 0.354 (red line); the expected mean value is at 0.50 for random structure pairs (magenta line). (C) The similarity score ratio for each miRNA duplex. Many miRNA duplexes were paired with multiple target bindings and thus received multiple similarity scores; the top 200 miRNA duplexes are shown here and are ordered by the count of scores received. Green: similarity score ≥0.7 and considered similar; Purple: similarity score <0.7 and considered different. Note the Y-axes on the four subgraphs were not drawn to the same scale. (D) Overall similarity ratios. Left: among the 17919 structure pairs considered, 69% are similar with RNAforester scores ≥0.7; Right: among the 249 miRNA duplexes that received more than 1 similarity scores, 78% have more scores ≥0.7, 14% has more scores <0.7 and 8% have equal counts.

To study if the distinctive base-pairing distributions of miRNA duplexes and miRNA-target binding structures hold at individual miRNA level, we systematically compared the 17919 pairs of miRNA duplex and miRNA-target binding structure that share a common guide strand. The RNAforester software was applied on the 17919 pairs to obtain the relative similarity score between the two members in each pair. Remarkably, nearly 69% of the pairs (12006/17919) have high similarity scores above 0.7 in a range of 0–1 (Figure 4A). The score cutoff value 0.7 was noted by RNAforester authors to indicate significant structure similarity [38]. In each of the 12006 pairs with high scores, the miRNA duplex structure is similar to its miRNA-target binding structure (Figure 4D, left). A closer way to look at this phenomenon is to individually consider the top 200 guide strands from the selected 321 miRNA duplexes that have the most number of targets, thus they have received the most number of structure similarity scores. Figure 4C reveals that most of their similarity scores are above 0.7 (green color). Among all 249 guide strands of the selected 321 miRNA duplexes that have received more than one similarity scores, 78% have received more scores above 0.7 than below, and only 14% have received more scores below 0.7 than above (Figure 4D, right). This indicates that miRNA-target binding structures generally mimic the associated miRNA duplex structure sharing the same guide strand.

**Figure 5:**
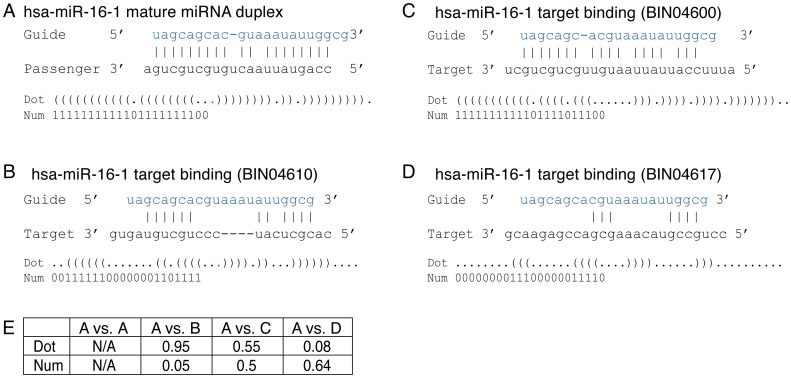
Examples of RNA structure encodings. (A) The miRNA duplex hsa-miR-16-1 and its corresponding dot-bracket and number notations. (B-D) The miRNA-target binding structures of hsa-miR-16-1 with 3 different target genes and their corresponding dot-bracket and number notations. (E) The similarity score and distance value between hsa-miR-16-1 duplex structure and each of its 3 miRNA-target binding structures that are calculated separately using the dot-bracket and number notations. The similarity scores and distance values visually correspond well to the perceived structure similarities in this example.

We have developed an alternative method to compare the structure similarity within a pair of miRNA duplex and miRNA target binding structure that share the same guide strand. In this novel method, the guide strand match pattern in each structure was converted into a series of numbers: matched bases were set to 1 and unmatched bases were set to 0 (Figure 5). As a result, two series of binary numbers were generated from each pair of structures. Subsequently, we calculated the length-scaled Hamming distance between these two series of numbers: distance values closer to 0 indicate the two series are very similar, distance values closer to 1 indicate the two series are almost exactly opposite, and distance values near 0.5 indicate the two series are likely unrelated. The mean of the 17919 distance values is 0.354 (Figure 4B, red line), and the 1^st^ quartile and 3^rd^ quartile are at 0.272 and 0.435 respectively. Z-test was applied to test whether 0.354 is significantly different from the expected mean value of 0.50 if the two number series in all 17919 pairs were completely random and unrelated to each other (Figure 4B, magenta line). The Z-test revealed a p-value less than 0.0001, thus we can conclude that most paired structures share a similar pattern and this similarity is not random. Taken together, our data suggest that the two types of structures, a miRNA duplex and its associated target binding structures, tend to be very similar when compared individually.

### The high pairwise structural similarity is unrelated to miRNA evolution

**Figure 6:**
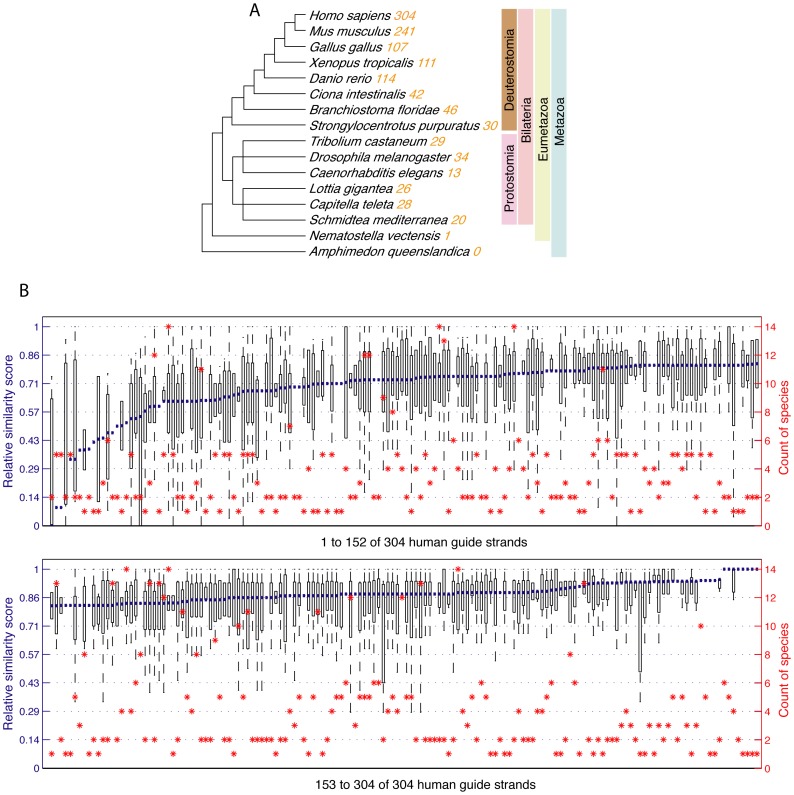
Correspondence of structure similarity to miRNA evolution. (A) The 321 selected miRNA duplexes contain 304 distinct guide strands; their homolog counts in each of the other 15 species is listed. (B) The similarity scores received by each miRNA guide strand are drawn in a boxplot with the median value identified by a blue line; the guide strands are then ordered according to their medians. The number of the other species that contain homologs to each human guide strand is also identified by a red dot. There is no discernable correspondence between the similarity score distribution in blue lines and the evolutional ages of miRNAs in red dots.

miRNAs are ancient. In the animal kingdom, miRNAs were present at the dawn of metazoan [39]–[42]. Two miRNA expansions were observed at the basin of bilaterian lineage and vertebrate lineage [41]. With the advent of next-generation sequencing, more miRNAs were discovered from various species. These studies suggested that the number of miRNAs in each species is closely correlated with its morphological complexity [36], [37]. So far, more than one thousand miRNAs have been found in humans according to the data from miRBase [43]. Some miRNAs are highly conserved whereas others are younger — they emerged just recently [44]. A total of 304 unique guide strands can be identified from the 321 miRNAs involved in the 17919 pairs selected for structure comparisons (see Figure 2 and Materials and Methods). We have selected for analysis 15 representative metazoan species that are from shorter to longer evolutionary distances from the humans. The number of known homologs among the 304 human miRNA guide stands in each of the 15 species is summarized in Figure 6A. None of the 304 guide strands has homologs in *Amphimedon queenslandica*, and only one, hsa-miR-100, has homologs in both bilaterians species and *Nematostella vectensis* — this is consistent with previous studies [45]. Most human miRNA guide strands have homologs in the other four vertebrates: *Mus musculus*, *Gallus gallus*, *Xenopus tropicalis* and *Daniorerio*. The number of miRNA guide strands that have homologs in protostomia decreases, which is also consistent with the notation that when some miRNAs became important in a particular lineage, they are rarely lost in the descendant lineages [39], [46].

When the 304 human miRNA guide strands are individually considered, about 85% of them have homologs in less than 6 other species (Table S5). The logical question to ask is whether the conclusion we have drawn in the previous section, that a miRNA duplex and its associated target binding structures tend to be similar, is only applicable to younger miRNAs? To answer this question, the similarity scores of the 17919 pairs of miRNA duplex and miRNA-target binding structures were mapped onto the 304 unique guide strands, which are then ordered and summarized in Figure 6B. For guide stands that received multiple mapped similarity scores, their score distributions are represented by boxplots and their median values are used in the ordering which goes from the least similar to the most similar (blue lines). Superimposed on Figure 6B is the count of the other species that have homologs to each of the ordered 304 human miRNA guide strands (red dots). From this figure it can be concluded that the similarity between miRNA duplex and miRNA-target binding structure is not correlated to miRNA evolution age.

## Discussion

The traditional understanding about miRNA is that the passenger strand is excluded during duplex unwinding and only the guide strand is needed to guide the activated RISC to target mRNAs; the passenger strand does not seem to play any role in the targeting process. However, recent studies in plants showed that the passenger strand may affect miRNA-triggered transitivity, a production of secondary siRNAs through antisense transcription [47],[48]. Further study revealed that the asymmetry in miRNA duplex structure is actually triggering the transitivity and the asymmetry is determined by both the passenger and guide strands [49]. These novel observations contradict the traditional guide-strand-centric understanding and suggest that passenger strands can still be functionally important before being excluded from RISC. Our data reveal that the miRNA-target binding structure mimics the miRNA duplex structure when they share a common guide strand. Therefore, we conclude that the passenger strand sequence, which helps determine the miRNA duplex structure, must also be considered in order to more precisely predict the targets of miRNAs at the structural level. Our conclusion coincides with the notation that the passenger strand may also be functionally important.

Target recognition is a binding process between the miRNA guide strand and targets. It is initialized by base-pairings in the seed region of the guide strand. Subsequently, compensatory base-pairings in the 3′ region of the guide strand, especially in the 12–17th locations, may enhance target binding [1], [50], [51]. In Figure 3B, the base-pairing distribution reveals that the seed region has both canonical and non-canonical matches. The high frequency of base-pairings in the non-seed region suggests that matches in this area may still be critical in target binding. On the other hand, the base-pairing distribution of miRNA duplexes (Figures 3A) reveals that they have a higher base-pairing frequency both in the seed region and the non-seed region. We can conclude from the global feature analysis that the base-pairing distributions of miRNA duplexes and miRNA-target binding structures are somewhat similar but they are not a simple mirror image as suggested in the literature [18].

Furthermore, tens of thousands of pairwise comparisons between miRNA duplexes and miRNA-target binding structures that share a common guide strand lead to a conclusion — the secondary structures of miRNA duplex and miRNA-target binding closely resemble each other (Figure 4). We can hypothesize that there are certain structural preferences of each miRNA guide strand which are carried forward from its binding with the passenger strand to its binding with the targets. The structural preferences are likely due to the physiochemical properties of the guide strand rather than evolutionary conservation because similar preferences can be observed from miRNAs of vastly different ages (Figure 6).

Our rationale is that miRNAs must form stable duplexes for Dicer cutting and RISC incorporation. Therefore, they require more stable overall base-pairing distribution as exhibited in Figures 3A. If too many mismatches happened anywhere on a duplex, the pre-miRNAs would not form a stable double-strand and that might have prevented mature miRNA production. When the guide strand binds to mRNA targets, it may encounter two possibilities: it may either bind more specifically to its targets with enhanced base-pairing in its 3′ non-seed region, or it may bind to more targets by tolerating more mismatches in this region. From our data, it seems that both possibilities are evident: the base-pairing distribution in the 3′ non-seed region is more dispersed as seen in Figure 3B, but the individual target binding structures are also specific, i.e., they mimic the miRNA duplex structures as seen in Figure 4. The guide strand may have certain physiochemical preferences to binding targets in its non-seed region that may enhance its potency. Also, non-specific guide strands might have interrupted too many genes and were quickly eliminated from the population during evolution.

We can draw the following conclusions from our study. First, it is not sufficient to consider just the guide strand sequence when predicting novel miRNA targets. The passenger strand, though previously considered irrelevant in miRNA target prediction, must also be considered to facilitate more precise miRNA target predictions at the structural level. Second, when binding to targets there are certain physiochemical preferences beyond the seed region of miRNA guide strands, which must also be considered to enhance miRNA target prediction precision. Taken together, our analysis on the pairwise structural comparison between miRNA duplexes and miRNA-target binding structures provides some new insights that may improve the precision of miRNA target predictions.

## Materials and Methods

### Structure encoding of miRNA duplex and miRNA-target binding

The secondary structures of miRNA duplexes were inferred using the hybrid-min software [52]. The miRNA-target binding data identified the guide strand of each miRNA. Oriented by the identified guide strand, the secondary structure of each miRNA duplexes was encoded into the dot-bracket and number notations (Figure 5A). The dot-bracket notation uses parentheses and dots to represent base-paired and mismatched bases; this notation was used by the RNAForester software to compare RNA structure similarities [53]. The number notation uses 1 and 0 to represent base-paired and mismatched bases; this notation was used in the Hamming distance calculation followed by the Z-test to also compare RNA structure similarities. There is a difference between the two notations: the dot-bracket notation encodes both strands of a miRNA duplex but the number notation encodes only the guide stand. The miRNA-target binding structures from the paper of Helwak *et al.* [30] were also determined and encoded into the dot-bracket and number notations in the same way (Figures 5B-D).

The Perl program mirna_structure_notations_v2.pl was created to automatically encode the structures of miRNA duplexes and miRNA-target bindings into the dot-bracket and number notations. Usage instructions can be obtained by running this program without any command-line argument. This program can also produce a global summary matrix counting the base-paired locations on the guide stand and passenger strand from all input structures. For example, if the 3rd base on the guide strand is base-paired with the 4th base on the passenger strand in a particular input structure, then the count at matrix location (3,4) will be increased by 1. Such matrices were used to create Figure 3.

### Pairwise comparison of miRNA duplex and miRNA-target binding structures

RNAforester is one of the structure comparison methods we have used [53]. This software compares two RNA secondary structures in the dot-bracket notation and determines their similarity score [53]. The Perl program mentioned above can convert the structures of miRNA duplexes and miRNA-target bindings to this notation. However, RNAforester only compares single-strand RNA foldings, not RNA duplex structures. Therefore, structures such as miRNA duplexes or miRNA-target bindings need to be converted into single-strand RNA folding representations. This is customarily accomplished by connecting together the dot-bracket notations obtained from the two strands of a duplex structure with a minimum of 3 dots to mimic a loop between them (see Figure 5) [54]. In our tests, we have found that the RNAforester similarity score is not sensitivity to the length of the loop (data not shown). To execute RNAforester solely for RNA secondary structure comparisons without also considering the primary sequence similarities, its parameter **bm** (base match score), **br** (base mismatch score) and **bd** (base indel score) were all set to 0, whereas its parameters **pm** (pairing match score) and **pd** (pairing indel score) were both set at the default values 10 and -5, respectively.

The miRNA duplex and miRNA-target binding structure in each selected pair share a common guide strand. The shared guide strand can serve as an *anchor* to determine the similarity between the two RNA secondary structures. We have created a novel method that can also determine RNA structure similarity. The Perl program mentioned above can convert the secondary structures of miRNA duplex and miRNA-target binding into a series of binary numbers. On the guide strand, a base-paired location is converted to 1 and an unmatched location is converted to 0. Subsequently, we calculated the length-scaled Hamming distance between the two series of numbers. If the two numbers on the same location is the same, 0 is added to the sum; otherwise 1/*n* is added to the sum, where *n* is the length of the guide strand. The sum of all values represents the distance between the two series of binary numbers. If the two series of numbers are the same, their distance should be 0. However, if they are complete reversals of each other, their distance should be 1. Any two random series should have an expected distance close to 0.50 because at each location there is equal probability to add or not to add 1/*n* to the sum, so the sum should approach 1/*n*×*n*/2 = ½. We have obtained 17919 distance values from the 17919 structure pairs. The mean of our distance values is 0.354, and the 1^st^ quartile and 3^rd^ quartile are at 0.272 and 0.435 respectively. This distribution is determined to be significantly different from the expected mean value of 0.50 for completely unrelated structure series after performing a Z-test (p-value<0.0001).

### The relationship between structure similarity and miRNA evolution

To determine if the similarities of miRNA duplexes and miRNA-target binding structures are related to miRNA evolutionary age, homologs to human miRNAs from the other 15 species were also selected from miRBase [55]. The miRNA names reflect their association to specific miRNA families. For example, the four miRNAs dre-miR-21 (*Daniorerio*), gga-miR-21 (*Gallus gallus*), mmu-miR-21 (*Mus musculus*) and hsa-miR-21 (*Homo sapiens*) all belong to the miR-21 family. We took a human miRNA-centric approach for the evolution study and considered only miRNAs from the other species that can be associated to the 321 human miRNAs selected for our study. Because only the guide strand is shared between a miRNA duplex and its miRNA-target bindings, we considered only guide stands in the evolution study. Some consolidation of guide strand data was performed. If two human miRNAs share the same guide strand, only one guide strand was retained for the study. Conversely, if both strands of a miRNA duplex are functional guide strands, both were retained for the study. For example, the two strands *hsa-let-7a-5p* and *hsa-let-7a-3p* of the human miRNA hsa-let-7a-1 are both guide strands, but they have different homologs in the other 15 species — 13 other species contain *hsa-let-7a-5p* homologs but only 5 other species contain *hsa-let-7a-5p* homologs.

After the consolidation, 304 distinct human miRNA guide strands were retained for the evolution analysis. The non-human miRNAs were initially assigned to the 304 human guide strands based on their name. If a non-human miRNA can be assigned to different human guide strands, they will be assigned in duplicate to simplify the analysis. Because some miRNA strand names are incomplete or incorrect, all non-human miRNAs assigned to a human guide strand were subsequently sequence-aligned to the guide strand to validate the assignment. If a non-human miRNA aligned poorly to the assigned guide strand, it was manually inspected and its assignment was removed when necessary. The counts of human miRNA homologs that can be found in the other species are listed in Table S5.

## Supporting Information

Table S1 547 mature miRNA duplexes selected from miRBase that have both strand sequences. (XLSX)

Table S2 18502 validated miRNA-target binding structures that were collected from the paper of Helwak *et al.*
(XLSX)

Table S3 321 unique miRNA duplexes from the intersection of the set of 547 miRNA duplexes having both strand sequences and the miRNAs involved in the set of 18502 validated miRNA-target binding structures. (XLSX)

Table S4 Dot-bracket and number notations for 17919 pairs of miRNA duplex and associated miRNA-target binding structure. Note that the two structures in each pair share a common guide strand. Also listed in the last two columns of the table are the RNAforester similarity score and the Hamming distance value for each pair of structures. (XLSX)

Table S5 The main part of the matrix listed the number of homologs of the 304 distinct human miRNA guide strands that can be found in each of the other 15 species; the 15 species were ordered according to their evolutionary distance from humans. The total number of human miRNA guide stands that have homologs in each species is summarized on the right side of the table under the column “Count of human siRNAs having homologs in each species”. The number of species covered by each of the 304 distinct human miRNA guide strand is also summarized at the bottom row of the table labeled “The number of species covered”. (XLSX)

Program S1 **Perl Program (downloadable Perl code).** The Perl program mirna_structure_notations_v2.pl used to generate the dot-bracket and number notations on Table S4. (PL)
